# Nur77 Serves as a Potential Prognostic Biomarker That Correlates with Immune Infiltration and May Act as a Good Target for Prostate adenocarcinoma

**DOI:** 10.3390/molecules28031238

**Published:** 2023-01-28

**Authors:** Qiong-Ying Hu, Jie Liu, Xiao-Kun Zhang, Wan-Ting Yang, Yu-Tian Tao, Ce Chen, Ye-He Qian, Jin-Shan Tang, Xin-Sheng Yao, Ying-He Xu, Jing-Hui Wang

**Affiliations:** 1 Taizhou Central Hospital (Taizhou University Hospital), School of Medicine, Taizhou University, Taizhou 318000, China; 2 Department of Pharmacy, Hebei Key Laboratory of Neuropharmacology, Hebei North University, Zhangjiakou 075000, China; 3 School of Pharmaceutical Sciences, Fujian Provincial Key Laboratory of Innovative Drug Target Research, Xiamen University, Xiamen 361102, China; 4 School of Integrated Chinese and Western Medicine, Anhui University of Chinese Medicine, Hefei 230038, China; 5 Institute of Traditional Chinese Medicine and Natural Products, College of Pharmacy, Jinan University, Guangzhou 510632, China

**Keywords:** prostate cancer, Nur77, prognosis, target

## Abstract

Prostate adenocarcinoma (PRAD) is the most frequent malignancy, and is the second leading cause of death due to cancer in men. Thus, new prognostic biomarkers and drug targets for PRAD are urgently needed. As we know, nuclear receptor Nur77 is important in cancer development and changes in the tumor microenvironment; whereas, the function of Nur77 in PRAD remains to be elucidated. The TCGA database was used to explore the Nur77 expression and its role in the prognosis of PRAD. It was shown that Nur77 was down regulated in PRAD, and low Nur77 expression was correlated with advanced clinical pathologic characteristics (high grade, histological type, age) and poor prognosis. Furthermore, key genes screening was examined by univariate Cox analysis and Kaplan-Meier survival. Additionally, Nur77 was closely related to immune infiltration and some anti-tumor immune functions. The differentially expressed genes (DEGs) were presented by protein-protein interaction (PPI) network analysis. Therefore, the expression level of Nur77 might help predict the survival of PRAD cases, which presents a new insight and a new target for the treatment of PRAD. In vitro experiments verified that natural product malayoside targeting Nur77 exhibited significant therapeutic effects on PRAD and largely induced cell apoptosis by up-regulating the expression of Nur77 and its mitochondrial localization. Taken together, Nur77 is a prognostic biomarker for patients with PRAD, which may refresh the profound understanding of PRAD individualized treatment.

## 1. Introduction

Prostate adenocarcinoma (PRAD) is the most frequent malignancy in male adults [1] and the second leading cause of death due to cancer in men [2]. In the past decade, although much progress has been made in the understanding of PRAD, due to the molecular heterogeneity of PRAD [3] and heterogeneous clinical responses to those therapies, an instant need for disease- and stage-specific molecular markers to accelerate accurate judgment on diagnosis, prognosis, and treatment response of PRAD is required. 

Nurr77 (also known as NR4A1, NGFI-B, TR3, and NAK), belonging to the nuclear hormone receptor superfamily, the NR4A subfamily [4,5], plays an important role in DNA repair, metabolism, tumor genesis, and inflammation [4,5,6]. Nur77 is correlated with several malignancies (including PRAD) which have been intensively investigated [7,8,9]. In PRAD, Nur77 mediates IGFBP-3-induced apoptosis [9] and inhibits androgen deprivation-induced cell invasion [10]. Overexpressed Nur77 in LNCaP cells restrains the proliferation and cell cycle [11]. The tumor immune microenvironment (TIME) comprises an abundance of inflammatory and immune cells, which is important for regulating tumor genesis and disease advancement [12]. Numerous studies provide attractive evidence that Nur77 can mediate inflammatory responses and affect the immunity to impact the course and outcome of cancers. Nur77 is a master regulator for Ly6C-monocytes and modulates the inflammatory effect of monocytes and macrophages [13,14]. Apoptosis mediated by Nur77 affects thymocyte selection and mature T-cell differentiation. Nur77 also has an effect on T cell tolerance and exhaustion [15,16,17,18,19]. Generally, Nur77 may act as a hopeful biomarker for PRAD prognosis prediction. However, Nur77′s clinical application value in PRAD remains largely unexplored. The relationship of Nur77 and infiltrating immune cells is required to be comprehensively analyzed in PRAD. The purpose of the present study is to systematically and comprehensively research the roles of Nur77 in PRAD and its potential relationship with clinic pathologic parameters by using data from several authoritative databases. Distinctive genomic alterations and functional networks which are relevant to Nur77, were also studied. The role of Nur77 in tumor immunity was also evaluated. 

Malayoside is a kind of cardenolide glycoside which is extracted from the Chinese herb *Antiaris toxicaria Lesch (Antiaris, Moraceae)* [20,21]. Our latest research suggests that malayoside inhibits non-small cell lung cancer cells (NSCLC) by modulating Nur77 [21]. We found that malayoside could also inhibit PRAD cells in the current study. Network pharmacological analysis, molecular docking, and molecular dynamics simulation analysis show that Nur77 was, moreover, a feasible target of PRAD treatment by malayoside. Next, through network pharmacology, gene ontology (GO), and Kyoto Encyclopedia of Genes and Genomes (KEGG) biological pathway functional enrichment, the target and target pathways of active components were studied. Malayoside showed a better correlation with MAPKs such as AKT1, EGFR, MAPK1, and SRC, which are important for Nur77 regulation. The top of the underlying mechanism of malayoside in PRAD was verified in vitro. In conclusion, our study may provide more direct evidence for assuming Nur77 as a potential predictive biomarker for patients with PRAD and for regulating Nur77 as a therapeutic target for PRAD.

## 2. Results

### 2.1. Nur77 Expression Was Downregulated in PRAD 

**Figure 1:**
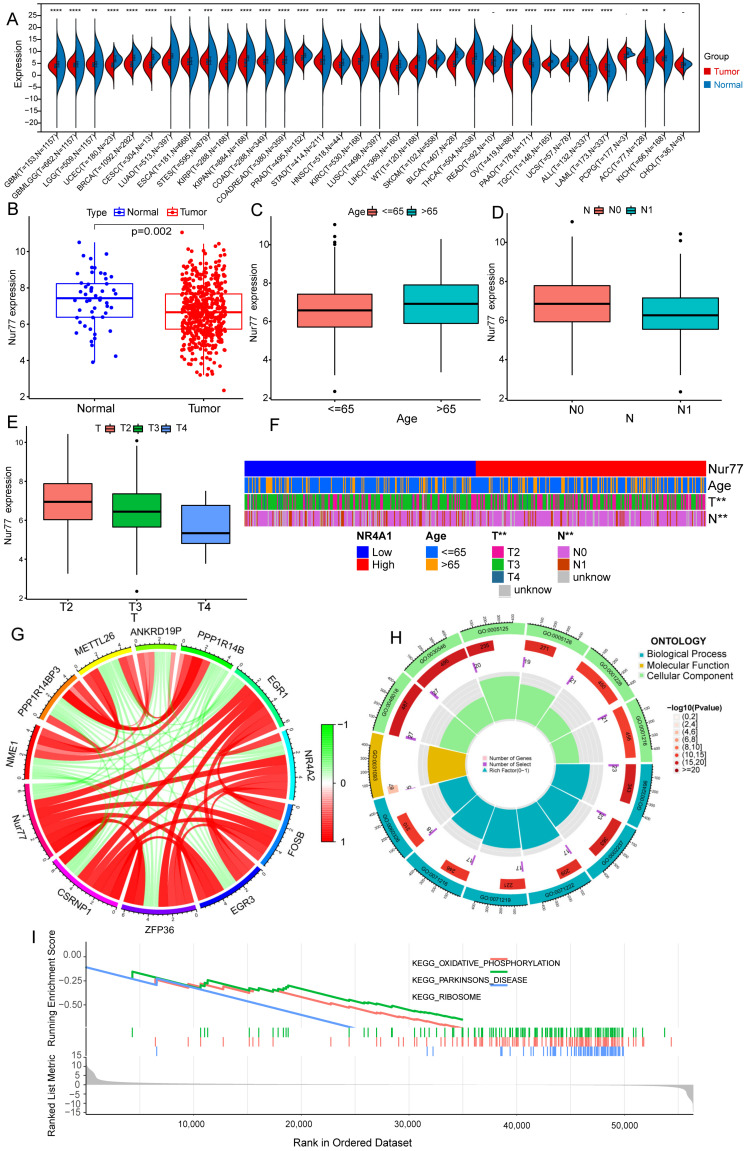
The Nur77 expression is associated with PRAD and clinicopathological features. (**A**) The Nur77 expression patterns of multiple kinds of tumors. (**B**–**E**) The scatter diagram showed that PRAD (**B**), age (**C**), Lymph node metastasis (**D**), and tumor development (**E**) were significantly associated with Nur77 expression. (**F**) Correlation between the expression levels of Nur77 and the clinicopathological features including age, Lymph node metastasis, and tumor development. (**G**) Co-expressed genes with Nur77, the red color represented a positive correlation, while the blue color indicated a negative correlation. (**H**) Gene ontology and (**I**) GSEA enrichment analysis of differently expressed genes in Nur77 high- and low- expression samples. (****, ***, **, * represent 0.0001, 0.001, 0.01 and 0.05).

To uncover the association between Nur77 expression and cancer, we surveyed Nur77 expression levels in 34 kinds of tumors, which indicated a significantly downregulated expression in the major kinds of tumors, including PRAD (Figure 1A,B). The investigation was then implemented to analyze the connection between Nur77 expression levels and clinical pathological characteristics (Figure 1C–F). The result showed that Nur77 was significantly more lowly expressed in PRAD patients with age below 65 than above 65 (Figure 1C) in the N1 stage than the N0 stage (Figure 1D), and in the T4 stage compared to the T2 and T3 stage (Figure 1E). A co-expression analysis revealed that Nur77 expression was positively correlated with EGR1, Nur77, FOSB, EGR3, ZFP36, and CSRNP1, as well as negatively correlated with NME1, PPP1R14BP3, METTL26, ANKRD19P, and PPP1R14B (Figure 1G). To further detect Nur77-mediated biological processes and cancer-associated pathways, we performed a Gene ontology and KEGG enrichment analysis of expression level differently regulated genes with Nur77, the findings of which were considerably enriched in cell chemotaxis, cellular response to biotic stimulus, cellular response to molecules of bacterial origin, cellular response to lipopolysaccharide, response to molecules of bacterial origin, and response to lipopolysaccharide (Figure 1H,I).

### 2.2. Nur77 Expression Correlated with Immune Cell Infiltration and Tumor Immune Microenvironment 

**Figure 2:**
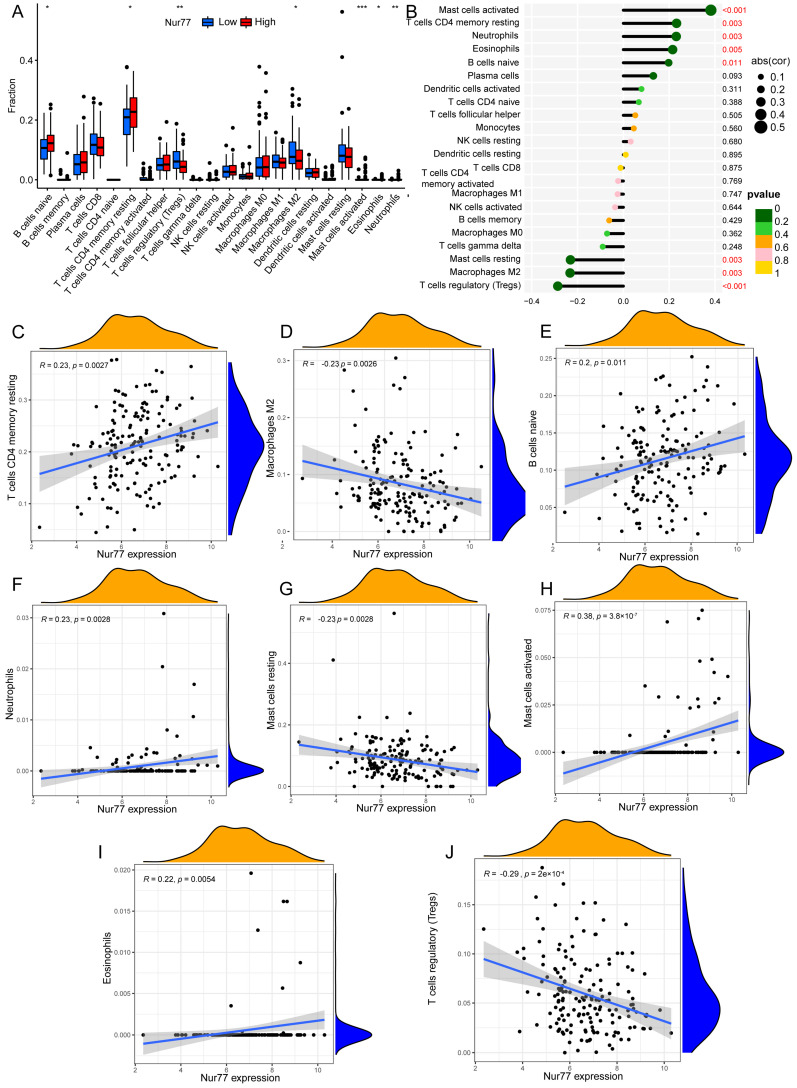
The association of Nur77 with tumor immune microenvironment and immune status. (**A**) The abundance of tumor-infiltrating immune cells in the high-Nur77 and low-Nur77 expression groups. (**B**) The correlation analysis between Nur77 expression and immune cell infiltration. (**C**–**J**) The correlation between Nur77 expression and T cells CD4 memory resting (**C**), M2 macrophages (**D**), naive B cells (**E**), neutrophils (**F**), resting mast cells (**G**), activated mast cells (**H**), eosinophils (**I**), regulatory T cells (Tregs) (**J**). (***, **, * represent 0.001, 0.01 and 0.05).

To discover the immune infiltration difference of different immune cells and immune status between high and low expression groups of Nur77, the infiltration score was quantified of diverse immune cell subpopulations. The results showed the group with low Nur77 expression levels was associated with many tumor-infiltrating immune cells, including CD8^+^ T cells, regulatory T cells (Treg), macrophages, and mast cells resting (Figure 2A). To further explore the potential relationship between Nur77 expression and immune infiltration, we performed an immune cell correlation analysis and discovered that Nur77 expression was positively correlated with mast cells activated, T cells CD4 memory resting, neutrophils, eosinophils, and native B cells, as well as those negatively correlated with mast cells resting, M2 macrophages, and regulatory T cells (Tregs) (Figure 2B–J). As shown in Figure 2C–J, we can see that *p* < 0.05, indicating that Nur77 and the immune cells are correlated. 

### 2.3. Functional Annotation and Pathway Enrichment of Nur77

**Figure 3:**
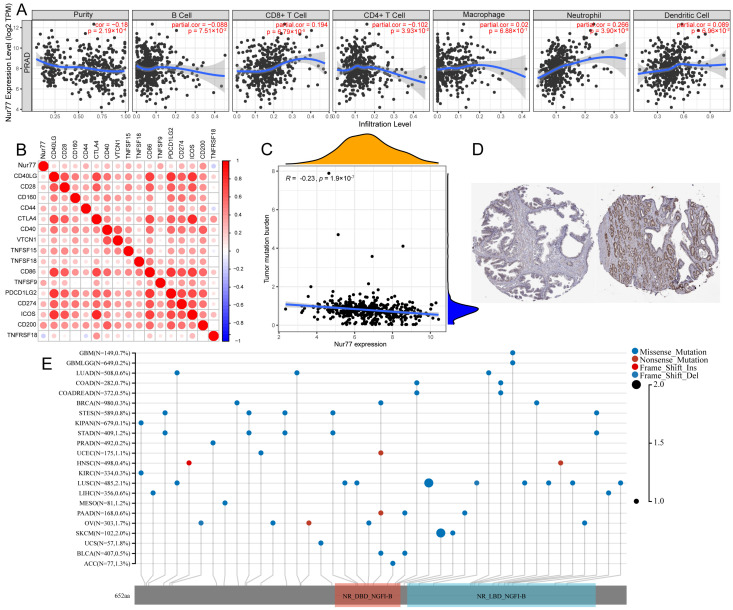
The correlation between Nur77 expression and immune cells in PRAD. (**A**) Nur77 expression was significantly associated with B cells, CD8^+^ T cells, CD4^+^ T cells, macrophages, neutrophils, and dendritic cells. (**B**) The correlation analysis of Nur77 expression and immune checkpoint. (**C**) The correlation between Nur77 expression and mutation status. (**D**) Representative images of immunohistochemical (IHC) staining of Nur77 protein in normal tissues and tumor tissue. (**E**) The mutations of Nur77 were presented in different kinds of tumors.

The role of Nur77 in immune infiltration revealed by TIME was correlated with previous findings that CD8^+^ T cells, macrophages, neutrophils, and dendritic cells were relevant to Nur77 expression, while B cell and CD4^+^ T cells were negatively correlated (Figure 3A,B). In addition, Nur77 expression was negatively correlated with the tumor mutation burden (Figure 3C). Apart from PRAD, 21 additional kinds of tumors also contain the Nur77 mutation, indicating that Nur77 may serve as a prognostic biomarker not only for PRAD patients, but it may also play functions in other kinds of tumors. This study revealed direct evidence for hypothesizing Nur77 as a potential predictive biomarker in PRAD patients and the modulation of Nur77 as a drug therapeutic target for PRAD. 

### 2.4. Induction of Apoptosis by Malayoside in LNCaP Cells

**Figure 4:**
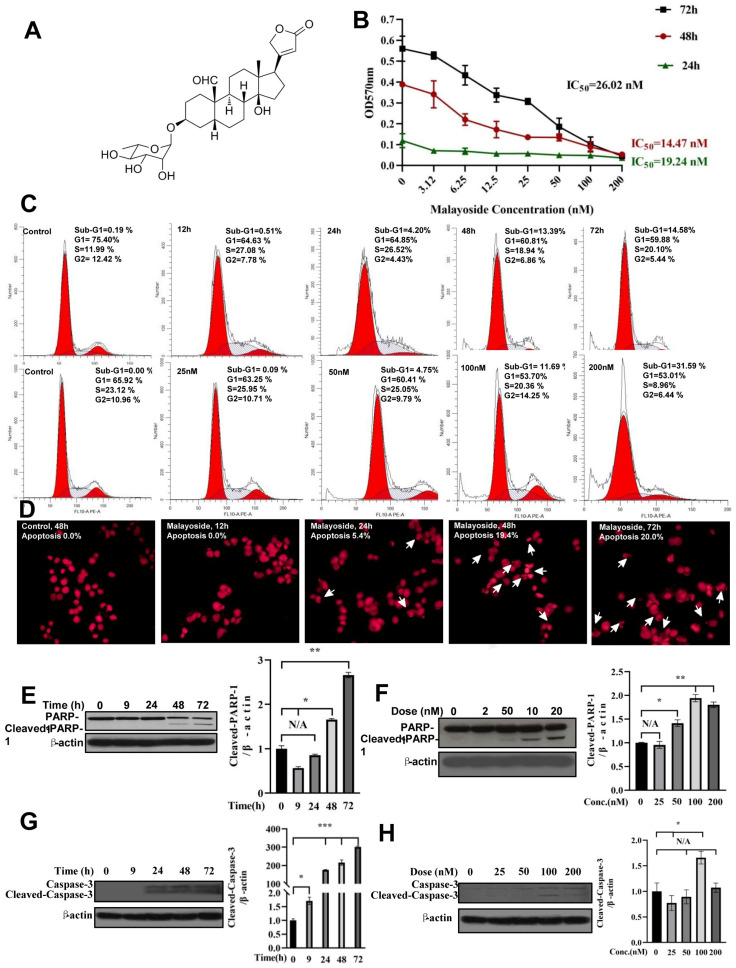
Induction of apoptosis by malayoside in LNCaP cells. (**A**). Schematic structure of malayoside. (**B**). Effect of malayoside on the growth inhibition of LNCaP cells for different incubation periods. Cells were treated with increasing concentrations of malayoside for 24, 48, and 72 h, respectively. Cell viability was measured by MTT assay. IC_50_ values are shown (n = 3). (**C**). Apoptosis induction was detected by flow cytometry (FCM) in LNCaP cells. LNCaP cells were treated at 100 nM of malayoside for 12, 24, 48, and 72 h (**top panel**), or treated at the indicated concentrations for 48 h (**bottom panel**). (**D**). Cell morphology was analyzed by PI staining in LNCaP cells. A nuclear fragment was seen in the cells treated with malayoside. Malayoside could induce PARP cleavage (**E**,**F**) and Caspase-3 cleavage (**G**,**H**). Cells were incubated with malayoside at the indicated time. All of the expression of apoptosis-related proteins was analyzed by western blot. Western blot bands were quantified and are shown in a histogram. The data are expressed as the mean  ±  SEM; * *p* < 0.05, ** *p* < 0.01, *** *p* < 0.001. The arrow indicates that nuclear fragmentation is observed in malayoside treated cells, suggesting that apoptosis occurs.

We found that malayoside (Figure 4A) modulated Nur77 to arrest NSCLC [21]. Here, we evaluated the growth inhibition effect of malayoside in LNCaP cells for different incubation periods by MTT assay (Figure 4B). Malayoside showed that the inhibition is in a dose-dependent manner no matter how long the incubation period is, with an IC_50_ value about 19 (for 24 h), 14 (for 48 h), and 26 (for 72 h) nM, respectively; along with an IC_90_ value greater than 1 μM (for 24 h), around 300 nM (for 48 h), and about 200 nM (for 72 h), respectively.

FCM and nuclear morphology analyses were performed to detect the apoptosis effect. The number of apoptotic cells (sub-G1) increased in a time-dependent manner (Figure 4C, upper panel). Treatments of 12 or 24 h only detected a small amount of apoptosis. After 48 h of treatment, the average apoptosis ratio was 13% when treated with 100 nM of malayoside. Furthermore, the apoptotic population was increased in a dose-dependent manner (Figure 4C, lower panel). When cells were treated at a 50 nM or lower concentration, an apoptosis ratio of less than 10% was detected; while at 100 nM, the cell apoptosis ratio increased to greater than 10%; moreover, when cells were incubated at 200 nM, the apoptosis ratio raised to 32%. In addition, PI staining showed that in LNCaP cells treatment for 72 h, morphological characteristics of apoptosis were significantly found (Figure 4D, *p* < 0.001), and apoptosis induction was also in a time-dependent manner.

Western blot analysis of Caspase-3 and cleavage of PARP were used to further investigate the effect on apoptosis. Malayoside induced cleaved PARP (83–89 kDa) in a time-dependent (Figure 4E) and dose-dependent fashion (Figure 4F). In addition, Caspase-3 was also activated by malayoside (Figure 4G,H). Thus, we determined that the malayoside could cause apoptosis in LNCaP cells.

### 2.5. Biological Enrichment and Network Analysis

The malayoside was then imported into the Discovery Studio 2017 to search for targets in two modes. In the shap mode, 34 targets were obtained; and in the no shap mode, 309 targets were acquired; in addition, 100 targets were gained in SwissTarget. After removing the duplicate values, 383 targets for the regulation of malayoside were achieved. The obtained PRAD-related targets were 2946, 683, 851, 107, and 19 by the GeneCards, DisGeNET, OMIM, TTD, and Drug bank databases, respectively. After deleting the duplicate, 3634 PRAD-related targets were gained. Finally, 230 common targets were obtained between malayoside and PRAD. 

**Figure 5:**
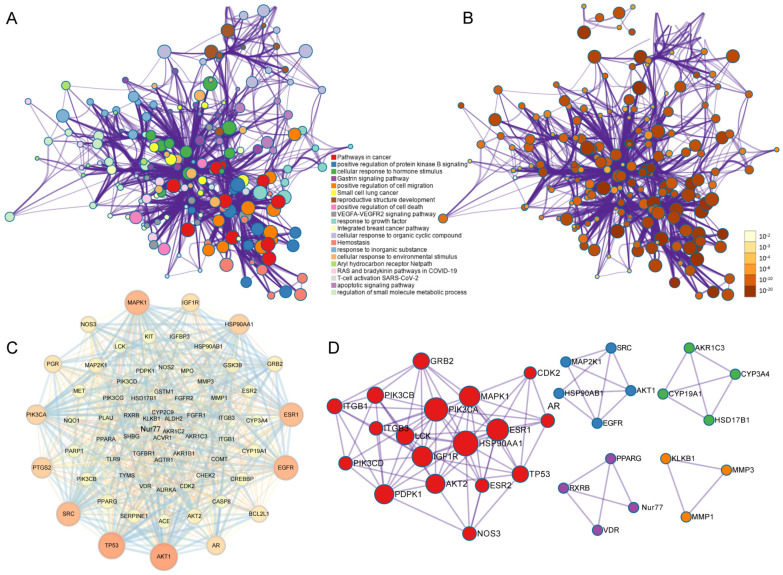
Biological enrichment and pharmacological network analysis. (**A**,**B**). Visual function enrichment analysis by Metascape. (**C**,**D**). PPI diagram of the common target network between Malayoside and prostate cancer.

To achieve a better understanding of the biological functions of these 230 common targets, Metascape software was utilized (Figure 5A,B). Figure 5B is another form of enrichment analysis. In fact, the depth of the color represents whether it is significant. The data showed that the enriched clusters were associated with PRAD. Moreover, after selecting the PRAD-related genes, we obtained 68 disease-related targets and protein-protein interaction (PPI) networks (Figure 5C,D). According to these analyses, the network contains 68 nodes and 639 edges, and the average number of nodes was 9.4. We found that the targets AKT1, TP53, EGFR, MAPK1, SRC, ESR1, AR, PTGS2, and HSP90AA1, HSP90AB1, and Nur77 were more important than other targets by using Cytoscape software to perform a topological analysis on the data. These proteins were involved in inflammation, cell proliferation, and apoptosis. This suggests that the above-mentioned targets might be crucial ones for the PRAD treatment effect of malayoside.

**Figure 6:**
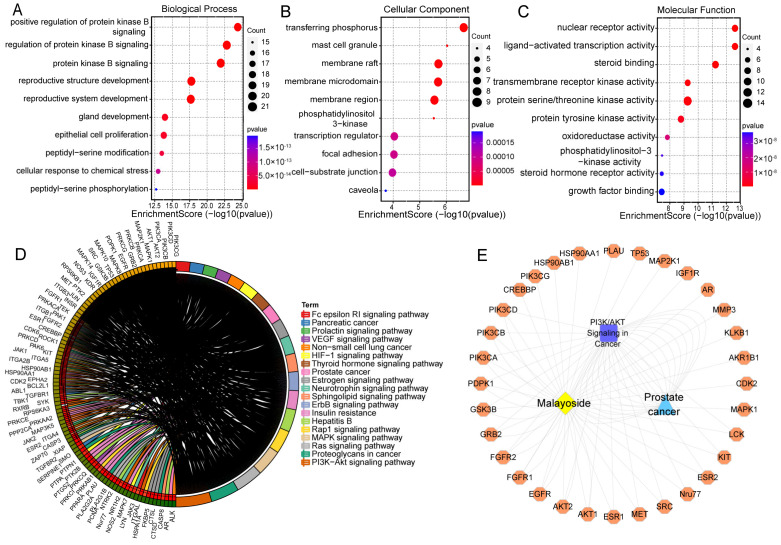
Pharmacological network analysis between malayoside and prostate cancer. (**A**–**C**). Go function enrichment diagram of the common targets of malayoside and prostate cancer. (**D**). KEGG pathway diagram of the common targets of malayoside and prostate cancer. (**E**). The compound-target-pathway-disease network of malayoside.

The GO function enrichment analysis of the common target of malayoside and PRAD was carried out through the DAVID database (Figure 6A–C). As a result, 340 GO entries were obtained (*p* < 0.05). Among them, the biological process (BP) entry was 228, the cell composition (CC) entry was 34, and the molecular function (MF) entry was 78, accounting for 67.06%, 10%, and 22.94%, respectively. The top 30 entries are shown in Figure 6A–C. Thus, the main function of malayoside was to regulate protein phosphorylation, cell migration, signaling transduction, as well as other functions to regulate the activities of various protein kinases existing in the cytoplasm, cell membrane, and exocytosis.

A total of 121 signaling pathways (*p* < 0.05) were obtained through the KEGG enrichment screening. We found that these pathways, including MAPK, PI3K-Akt, ErbB, and HIF-1 signaling pathways, were mainly associated with PRAD (Figure 6D). Potential targets and the corresponding active ingredient for the treatment of PRAD by malayoside were used to construct a traditional Chinese medicine-compound-target-disease network, and the data were imported into Cytoscape for visualization. As is shown in Figure 6E, the “compound-target-pathway-disease” network diagram suggested that malayoside affected PRAD by acting on 58 targets to regulate two signaling pathways closely related to PRAD that involve the MAPK and PI3K-Akt pathways.

According to our network pharmacology analysis described above (Figure 5 and Figure 6), we declared that the treatment of PRAD by malayoside was dependent on Nur77.

### 2.6. In Silico Validation of the Ligand-Target Interactions

We next clarified the accurate binding model between the Nur77 active site and malayoside in silico, in addition to predicting the dynamic changes of the system when given an initial model and conditions. Through GO enrichment analysis (Figure 6A–C), we found that these different targets are closely related to the corresponding biological processes, such as the positive regulation of protein kinase B signal, phosphatidylinositol 3-kinase and steroid hormone receptor activity, which are closely related to the pathogenesis of PRAD.

#### 2.6.1. Molecular Dynamics Simulation

**Figure 7:**
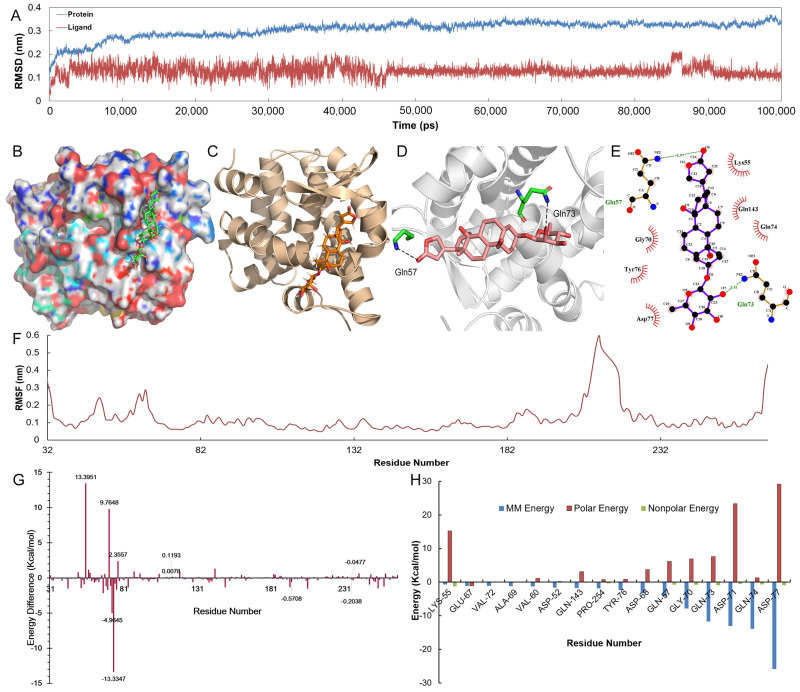
MD results. (**A**). The RMSD diagram for the initial structure of the protein and ligand during MD simulation. (**B**,**C**). Binding conformation of the ligand malayoside into the binding pocket of the Nur77. (**D**,**E**). The detailed view of the two-dimensional and three-dimensional binging interactions between malayoside and Nur77. (**F**). The RMSF of the malayoside-Nur77 complex system. (**G**). The decomposition energy diagram of each amino acid in the binding site. (**H**). MM energy, nonpolar and polar solvation energy of the key residues.

The molecular dynamics (MD) simulation with 100 ns was run based on the initial structure of the docked complex of Nur77 protein with malayoside to investigate the conformation sampling of the ligand into the binding pocket and further analyze the dynamics of the ligand-protein interaction. In addition, the root-mean-square deviation (RMSD) (Figure 7A) of the backbone atoms for the route was calculated to indicate the positional stability of the original complex shape. The outcome indicated that the structure of this complex acted reasonably stable after 2 ns, while the RMSD for the initial structure of the complex reaches about 0.3 nm after 2 ns and virtually maintained this value over the subsequent simulation. After MD simulations, the binding interactions of this complex were shown in Figure 7B–E. Intriguingly, the molecule binding mode matched the docking data, in which H-bonding interactions (two strong H-bond interactions with Gln57 and Gln73) were revealed to have a role in binding affinity with the Nur77 protein. Malayoside is embedded into binding sites created by residues Gln57, Gln73, Lys55, Gln143, Gln74, Gly70, Tyr76, and Asp77. 

The ligand moved considerably closer to the amino acids and binds extensively into the binding cavity as an outcome of these interactions, showing that the ligand matches well with the receptor’s binding site and that the complex could become permanent over a lengthy period of MD simulations. The root-mean-square-fluctuation (RMSF), a frequently used approach for evaluating the variation and movement of the protein backbone and side-chain residues that appear in MD simulations, was computed according to a reference structure that illustrates the mobility of residues in the binding protein. The hydrophobic channel residues exhibited the lowest RMSF value, indicating the reduction of structural rearrangements, deviation, and variance, while less internal mobility of the complex and the adaptability of these residues were found inside the catalytic sites (Figure 7F).

Nonetheless, the RMSF scores of backbone residues in the terminal and loop areas were in the range of 0.3–0.6 nm, indicating that these residues have substantial variations. The protein-ligand complex had robust structures based on the MD simulation, as almost all of the residues remained with an RMSF value at 0.1 nm in most of the trajectories, showing the rationality and validation of this docking model.

#### 2.6.2. The Binding Free Energy 

The binding free energy of chemical malayoside with protein Nur77 was determined using the G_MMPBSA tool to evaluate the activity of the therapeutic molecule (Table 1). As was shown in Table 1, the estimated total binding free energy of this complex system was −88.856 kJ/mol, indicating the malayoside’s high biological activity and demonstrating the ligand’s excellent binding affinity to the receptor. Additionally, the binding energy was split into individual residues to get more comprehensive interactions of this complex. Figure 7G,H showed the findings of decomposition energy for each essential amino acid in the active site. Clearly, residues of Gln57 and Gln73 played a significant role in the ligand’s binding to the Nur77 protein. These residues suggested the extensive interaction with malayoside, resulting in the complex system’s stable conformation and improved antagonistic activity. Because most of the key residues had hydrophobic characteristics, it was reasonable to conclude that the van der Waals interaction contributed most of the binding system’s energy.

Furthermore, the exploration of H-bonding revealed that residues Gln57 and Gln73 play critical roles in the molecular recognition of this complex. Additionally, Figure 7H demonstrated that the non-polar solvation energies of residues Asp77, Gln73, Gln74, Asp71, and Gly70 are the primary driving force for increasing the malayoside’s binding affinity to the Nur77 protein, highlighting the critical significance of hydrophilic interactions throughout the binding process. All of these findings showed that H-bonding, electrostatic and hydrophobic interactions dictate the binding affinities of these ligands and that our docking model is accurate.

### 2.7. Correlation between Malayoside Apoptotic Effect and Nur77 Expression

**Figure 8:**
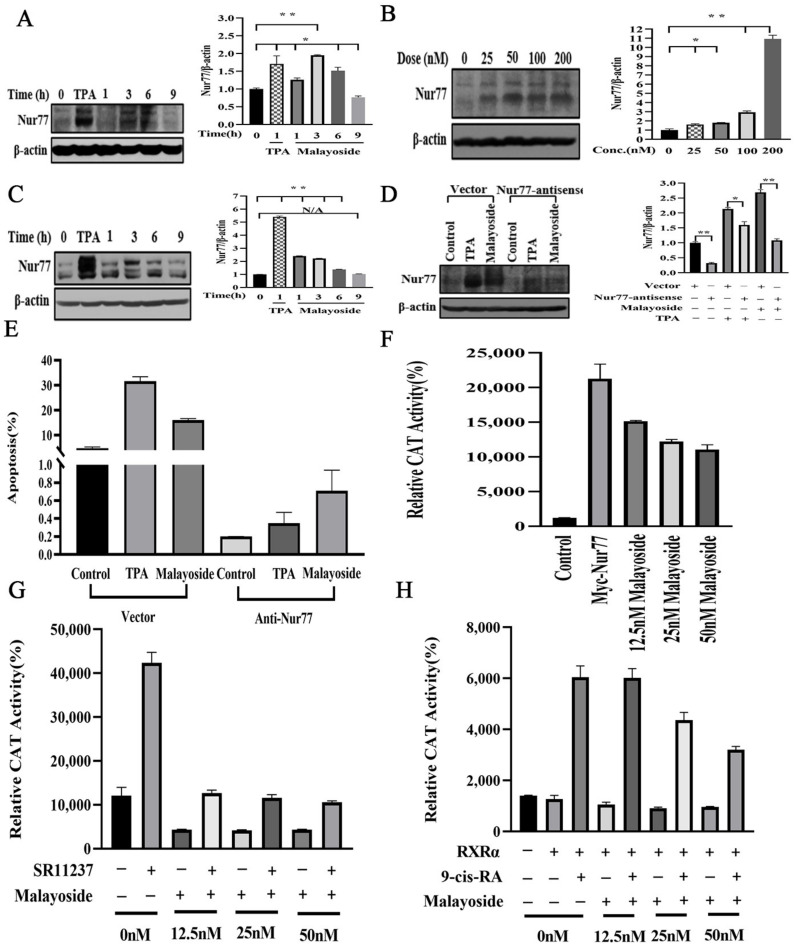
Correlation between apoptosis of malayoside and its induction of Nur77 expression. (**A**). Induction of endogenous Nur77 expression by malayoside in a time-dependent manner. LNCaP cells were treated at 100 nM of malayoside for 0, 1, 3, 6, and 9 h. (**B**). Induction of endogenous Nur77 expression by malayoside in a dose-dependent manner. LNCaP cells were treated at 25, 50, 100, and 200 nM of malayoside for 3 h, respectively. Bands were quantified and shown in the histogram. (**C**). Malayoside induced more Nur77 protein expression in Nur77 overexpressed LNCaP cells. LNCaP cells were transfected with vector or pCMV-Myc-Nur77 plasmid and treated with malayoside for hours as indicated. (**D**). Anti-sense Nur77 inhibited malayoside-induced Nur77 protein expression in LNCaP cells. All of the protein expression was detected by western blot. Western blot bands were quantified and shown in a histogram. The data are expressed as the mean  ±  SEM; * *p* < 0.05, ** *p* < 0.01. (**E**). Anti-sense Nur77 inhibited malayoside-induced apoptosis in LNCaP cells. Apoptosis was detected by FCM. The cells were treated with malayoside the same as in the previous experiment. (**F**,**G**). The inhibition of transcriptional activation of Nur77 by malayoside. LNCaP cells were transiently transfected as described in “Section 4”, treated with malayoside, and assayed for CAT activity after 24 h. (**H**). Inhibition of transcriptional activation of RXRα by malayoside. CV-1 cells were transiently transfected as described in “Section 4,” treated with malayoside, and assayed for CAT activity after 24 h.

We have reported that malayoside-induced apoptosis in NSCLC was associated with the inducible expression of Nur77 [21]. In PRAD cells, we first used the western blot to analyze Nur77 expression (Figure 8A–C). The outcome showed that in treatment of c malayoside for 3 h, Nur77 expression was significantly induced and maintained at a high degree over 6 h, but then dramatically declined to a non-treatment level at 9 h (Figure 8A). As a positive control, remarkable expression induced by (phorbol ester) TPA was detected (Figure 8A), which was consistent with the previous report [22]. Furthermore, Nur77 expression level gradually increased with dose when treated for 3 h (Figure 8B). Moreover, in Nur77-overexpressed LNCaP cells, malayoside, along with TPA, also induced more Nur77 expression (Figure 8C). For comparison, in Nur77-antisense RNA-expressing LNCaP cells, neither TPA nor malayoside could induce greater Nur77 expression (Figure 8D). In addition, Nur77 displayed multiple bands upon malayoside treatment (Figure 8A–D), suggesting its post-translational modifications. Nur77 was phosphorylated in several studies [23,24,25,26]. Vector-transfected and Nur77-antisense RNA-expressing LNCaP cells were used to evaluate apoptosis induction by malayoside. FCM results showed that the apoptotic effect of malayoside was attenuated for approximately 20% in Nur77-antisense RNA-expressing LNCaP cells (Figure 8E). As we have reported, overexpressed Nur77 in the H460 cells could induce more but not so apparent apoptosis [21]; therefore, we did not try to induce apoptosis in overexpressed LNCaP cells. Altogether, the apoptotic effect of malayoside in LNCaP cells is dependent on Nur77 expression.

A Nur77 homodimer binding site, NurRE, was activated during T-cell apoptosis [27,28]. To clarify whether the up-regulation of Nur77 expression and induction of apoptosis by malayoside was involved in Nur77 transactivation, a (Catalase) CAT reporter gene expression vector which contains a Nur77-binding sequence (NurRE) [28] was transiently transfected into LNCaP cells. Basal reporter gene activity was very low due to the lack of endogenous Nur77 (Figure 8F). When the exogenous Nur77 expression vector, together with the NurRE reporter gene expression vector, was cotransfected into LNCaP cells, malayoside treatment did not activate, but repressed the transcriptional activity of the reporter gene in a dose-dependent manner (Figure 8F). Therefore, the up-regulation of Nur77 protein was not correlated with its repressed transactivation activity when stimulated by malayoside. 

Previous studies have indicated that several orphan receptors, such as Nur77 [25,26] and COUP-TF [23,24,28], have been implicated in the regulation of retinoid responses. Furthermore, the βRARE can also bind Nur77/RXR heterodimers that are activated by RXR-selective ligands [29]. Consistent with other observations [30], we found that cotransfection of Nur77 with RXR expression vectors enhanced RARβ promoter activity in response to RXR-selective retinoid SR11237 (Figure 8G). Malayoside also inhibited the transcriptional activity of the reporter gene with or without SR11237 in a dose-dependent manner (Figure 8G). Thus, in LNCaP cells, the transactivation activity of Nur77 was repressed rather than enhanced, irrespective of the presence or absence of the Nur77 expression vector (Figure 8F–G). In addition, we further evaluated the effect of malayoside on RXR/RXR transcriptional activity. It was also seen that malayoside could not activate the RXR/RXR homodimer when treated alone, and 9-cis-RA-induced reporter activity was also reduced by it in a concentration-dependent manner (Figure 8H). Therefore, we could conclude that the up-regulation of Nur77 expression and the induction of apoptosis by malayoside were not involved in Nur77 transactivation. 

### 2.8. Role of Nur77 Cytoplasmic Translocation for Its Apoptosis Induction 

**Figure 9:**
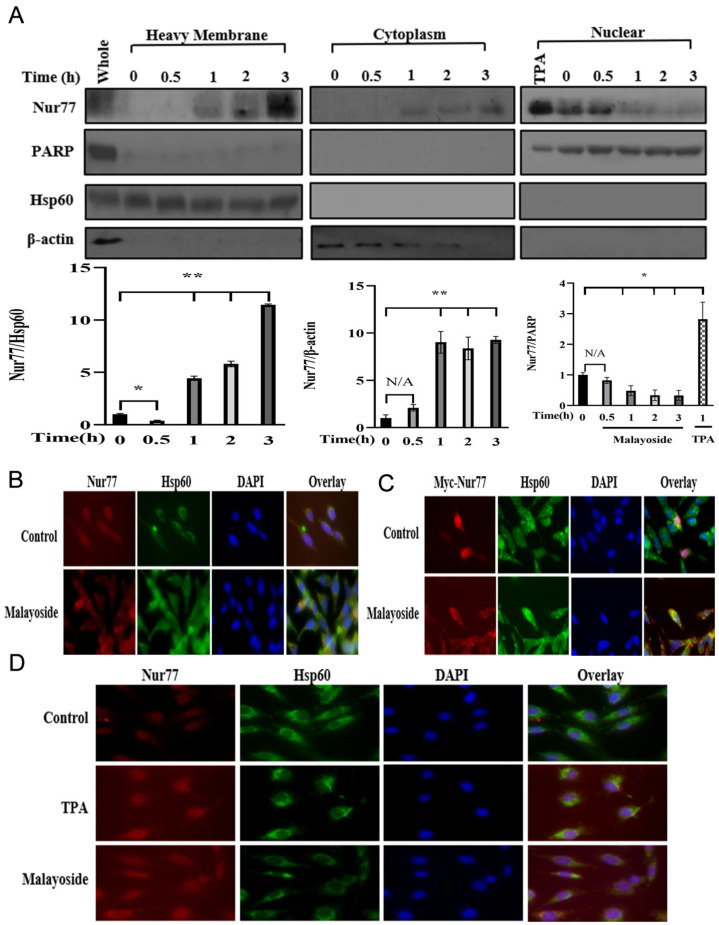
Malayoside could induce Nur77 nuclear export in LNCaP cells. (**A**). Cell fraction proteins were detected by western blot analysis. The exact location of Nur77 in a heavy membrane, cytoplasm, or nuclear depended on the incubation time of malayoside. Western blot bands were quantified and shown in a histogram. The data are expressed as the mean  ±  SEM; * *p* < 0.05, ** *p* < 0.01. (**B**–**D**). Nur77 nuclear export was measured by fluorescent immunostaining and florescent microscopy in wild-type, Myc-Nur77 overexpressed, and anti-sense Nur77 LNCaP cells, respectively.

It has been reported that the redistribution of transcription factors and kinases is important for the regulation of their activities and the execution of their functions; furthermore, phosphorylation of these proteins is necessary for such redistribution [31,32,33]. Because translocation of Nur77 from the nucleus to mitochondria plays a critical role in the regulation of apoptosis [34,35,36], we measured whether Nur77 redistributed after the treatment with malayoside (Figure 9). A cellular fractionation assay showed that Nur77 in the fraction of mitochondrion-enriched heavy membrane increased in a time-dependent manner, occurring as soon as 1 h after the treatment (Figure 9A). Such an accumulation of Nur77 at the mitochondria was accompanied with the decrease of it in the nucleus (Figure 9A); meanwhile, an enhancement of Nur77 was also detected in the cytoplasmic fraction after 1 h of treatment, indicating that Nur77 may also be effective in the cytoplasm other than the mitochondria. Interestingly, it appeared that Nur77 protein with slow migration (high-molecular-weight band) on (SDS-polyacrylamide gel electrophoresis) SDS-PAGE predominantly resided at the mitochondria, while the fast-migrating Nur77 band was primarily found in the nucleus after malayoside treatment, and slightly in the cytoplasm. Such an observation suggested that the phosphorylation of Nur77 may be required for the induction of Nur77 nuclear export by malayoside in the LNCaP cells. Coincidentally, mitochondrial localization was proved by immunostaining (Figure 9B). Thus, the malayoside-induced translocation of Nur77 resulted.

When Nur77 was overexpressed, Nur77 translocation significantly appeared by malayoside treatment in LNCaP cells (Figure 9C, *p* < 0.01), but the redistribution was eliminated or weakened by antisense Nur77 receptor transfection (Figure 9D), making it clear that Nur77 is important to the redistribution processes. Thus, the apoptosis induced by malayoside decreased in Nur77-antisense RNA-expressing LNCaP cells (Figure 9E), which was caused by not only by Nur77 expression, but also its redistribution.

## 3. Discussion

Prostate adenocarcinoma (PRAD) is the second most frequently diagnosed malignancy [37]. Its diagnostic rate is about 12%, accounting for 9% of male cancer deaths [38]. Prostate-specific antigen (PSA) has been used for PRAD screening for a long time, but the weaknesses of PSA, such as low sensitivity and accuracy, result in a high rate of overdiagnosis and overtreatment of PRAD [38]. Accurate prostate-specific biomarkers are needed.

Nur77 functions as a potent sensor of the cellular microenvironment, with the activities of controlling metabolism, vascular homeostasis, inflammation, and cancer. Numerous studies have shown that Nur77′s functional roles are associated with its expression levels and subcellular localization [8,39]. However, the expression and functions of Nur77 in PRAD have yet to be revealed. Here, we tried to understand the expression pattern, prognostic values, and functions of Nur77 in PRAD. We have identified that the gene expression of Nur77 was downregulated in PRAD tissues by using a bioinformatics analysis. Nur77 coincided with a poor prognosis in PRAD. We established a nomogram to study the function of Nur77 in PRAD. We integrated Nur77 with clinicopathological factors (age and grade), and found that Nur77 performs exceptionally well in predicting OS in PRAD patients. We also investigated the relationship between Nur77 and TIME via GSVA (gene set variation analysis). Nur77 is closely associated with immune infiltration in PRAD. It is known that TIME is important in the pathogenesis and progression of PRAD cells [40,41]. Multiple different immune cell types have been identified in PRAD. Immune cells are involved in the development and progression of PRAD. Most immune cells have the potential to either support or promote the growth of PRAD. Macrophages [42], neutrophils [43], and mast cells [44] mostly exhibit pro-tumor qualities, while high numbers of T cells (specifically CD4^+^ or CD8^+^ T cells) [45] or B cells [46] predicted worse capsular and perineural invasion. Our results showed that CD8^+^ T cells, macrophages, neutrophils, and dendritic cells were positively correlated with Nur77 expression, while B cells and CD4^+^ T cells were negatively correlated (Figure 3A,B). The deletion of Nur77 in TIME macrophages promotes TNF-α production. The deletion of Nur77 also reduced CSF-1R expression. The lack of CSF-1R results in the metastasis of cancer cells. In high levels of tolerant T cells, Nur77 expression is increased. IN T cells, the overexpression of Nur77 restrains effector T cell differentiation. The deletion of Nur77 overcomes T cell tolerance and exaggerates the effector function, as well as enhancing immunity against tumors [47]. The treatment effect of Nur77^−/−^ CD8^+^ T cells’ adoptive cell therapy (ACT) is better than wild-type CD8^+^ T cells [48]. Thus, Nur77 is essential for T cell tolerance and represses antitumor immunity. Nur77 expression also promotes CD^4+^ single-positive thymocytes differentiation into Treg cells. The lack of Nur77 in Treg cells shows resistance to tumor growth. In CD8^+^ cytotoxic T cells, the suppression of Nur77 expression can release the effector activities. In TIME, lacking Nur77 stimulated effective immune responses. 

The expression, function, and effect on Nur77 in PRAD cells and its effect on TMIE demonstrated that Nur77 could be a new therapeutic target for PRAD treatment and prevention. Malayoside is a cardenolide glycoside which is extracted from *Antiaris toxicaria Lesch* (Figure 4A). It has been reported that cardenolide glycosides have a dramatic anti-cancer role in diverse types of human cancers, such as PRAD [49,50]. The anti-cancer effect of cardenolide glycosides was associated with a variety of proteins and signaling pathways [51,52,53]. In the present study, malayoside was found to be critical to the suppression of the growth (Figure 4B) and induction of apoptosis (Figure 4C–H) in PRAD cells. We used network pharmacology to elucidate the possible targets and mechanisms by which malayoside relieved PRAD. A signaling pathway analysis suggested that malayoside may play its antitumor effect on PRAD through the adjustment to cell survival and death (Figure 5A). Signaling pathways that regulate numerous biological functions are commonly dysregulated in the pathological progression of PRAD. Through network pharmacology analysis, malayoside may inhibit prostate cancer by regulating PRAD cell proliferation and death by nuclear receptors (NR), MAPK, PI3K/AKT, TP53, Bcl-2, matrix metalloproteinases (MMPs), and their signaling pathways, especially with NRs and MAPKs. The KEGG results indicated that the nuclear receptors and MAPKs exhibited higher gene count enrichment among the cancer-related molecular function and biological process. Nur77 might be critical to controlling the anti-cancer action of cardenolide glycosides [21,54,55,56]. Nur77 mediates cancer cell apoptosis by several apoptotic stimuli, including chemotherapeutic agents [57,58]. Docking results demonstrated that malayoside binds Nur77 LBD at the binding pocket through two bonds (Figure 7). Moreover, the cellular experiments exhibited that malayoside induced Nur77 expression (Figure 8A–D) and mitochondrial localization in PRAD cells (Figure 9A–D). The apoptotic effect of malayoside was also associated with Nur77 (Figure 8E). Apoptotic agents induce the nuclear export of Nur77 [34,35,58,59,60,61,62], which associates with Bcl-2 conformational change [34,35,59,63,64,65,66]. It should be pointed out that Nur77 is a highly phosphorylated protein which can be phosphorylated by Akt and MAPKs at various amino acid residues in different kinds of cells [22,35,67,68,69,70,71]. By contrast, phosphorylation by Akt reduces Nur77-mediated apoptosis [22,69,70,71], while phosphorylation by Jun N-terminal kinase (JNK) promotes its cytoplasmic localization and apoptotic effect [22]. p38 MAPK and ERK also regulated the apoptotic effect of Nur77. For instance, CCE9 activates p38α MAPK to induce Nur77 expression and apoptosis [35]. We have uncovered that malayoside activates both p38 MAPK and ERK to regulate Nur77-dependent apoptosis in NSCLC cells [21]. Nur77 always interacts with Bcl-2 at mitochondria in the process of apoptosis. Binding Nur77 leads to the conversion of Bcl-2. Bcl-2 changed from a survivor to a killer [35,60,63,65]. MAPKs regulate Nur77 mitochondrial localization and the interaction with Bcl-2 [35,64,65,72,73,74,75,76]. Based on our network pharmacology data, malayoside seemed to particularly regulate ERK MAPK (MAPK1) and PI3K-Akt. The ERK pathway is critical to the multistep development of several human tumors, which relates to the cell proliferation, differentiation, and cell cycle, promotion of cell survival through Bcl-2 family proteins, and the contribution of cell invasion through MMP [77], while PI3K/Akt manages cell metabolism, growth, proliferation, and tumor development [78,79,80,81,82,83]. The PI3K/Akt pathway is a prominent alternate pathway in PRAD [84]. MAPK/ERK and PI3K/Akt are important in mature T cells. Both signaling pathways promote the phosphorylation of Nur77. 

## 4. Materials and Methods

### 4.1. The Acquisition of Compound Targets 

The Ligand Profile in Discovery Studio 2017 was used for reverse targeting. It was conducted in two modes: shap and no shap. The parameters had defaulted and the website of SwissTarget (http://www.swisstargetprediction.ch/, accessed on 3 May 2011) was used for the target search. The obtained drug targets were standardized by the UniProt database (https://www.uniprot.org/uniprot/, accessed on 1 January 2004).

### 4.2. Acquisition of Prostate Cancer Targets

Using “prostate cancer” as the keyword, we searched and screened disease-related disease targets through the OMIM (https://www.omim.org/, accessed on 2004), TTD (http://bidd.nus.edu.sg/BIDD-Databases/TTD/TTD.asp, accessed on 7 January 2022) and DisGeNET (https://www.disgenet.org/, accessed on 21 September 2010) databases. The disease targets and the compound targets were mapped to take the intersection and then the Venn diagram was drawn. We imported the common targets to Cytoscape 3.6.1 to construct the “Malayoside-Common Target-Related Pathway-Prostate Cancer” network.

### 4.3. Biological Enrichment and Network Analysis 

To construct a protein interaction network, the obtained key targets were imported into the Metascape database (https://metascape.org/gp/index.html, accessed on 8 October 2015). The conditions that were set as species (homo sapiens), minimum interaction score (medium confidence, 0.4000), and free protein spots were removed. We imported the obtained the protein interaction network into Cytoscape 3.6.1 and used the Network Analyzer module for visual processing and analysis. In addition, to obtain the function of the intersection gene and its role in the signaling pathway, we used the DAVID 6.8 platform (https://david.ncifcrf.gov/, accessed on 18 December 2008) to process the intersection target obtained by the screening. We performed a GO enrichment analysis by entering the name list and limiting the species to “homo sapiens” (adjusted *p*-value < 0.05), and selecting the top five entries. The GO analysis and PPI network construction were also conducted by Metascape [85,86].

### 4.4. Molecular Docking

The Protein Data Bank database (https://www.rcsb.org/, accessed on 2008 was used to determine the crystal structure of the Nur77 protein (PDB ID: 4RZG). Additionally, the Autodock Vina (Version 1.1.2) [87] was performed to execute molecular docking. We computed the binding affinities and prioritized the values to pick the optimal posture in each search space after a series of ligand-receptor docking operations. Subsequently, the docking findings were compared among activity pockets to determine which ligands had the best overall posture. 

### 4.5. Molecular Dynamics Simulation

The GROMACS 2019 software validation of this complex utilizes the ambersb99 force field [88]. The AutoDock docking conformation with the lowest binding energy (most negative) was used as the starting point for the MD simulation. Gromacs software was used to construct the topological parameters of proteins. The compound was placed in a box filled with water molecules with a simple point charge (SPC). To guarantee the overall charge neutrality of the simulated system, 7 Na^+^ counter-ions were introduced by substituting water molecules. The solute was subjected to a position-restrained dynamics simulation at 300 K for 200 ps to equilibrate the system. The entire system was put through its paces in an MD production run at 300 K and 4.5 × 10^−5^ bar^−1^ pressure using the Parrinello-Rahman scheme [89]. Finally, a long 100 ns run was performed to ensure the stability of the MD simulation.

### 4.6. Evaluation for Binding-Free Energy

The binding free energy for the complexes of Nur77 protein and malayoside chemical was estimated with the G-MMPBSA method based on the Molecular Mechanics/Poisson-Boltzmann Surface Area approach (MM-PBSA) [90]. The governing formula was used to measure the differences in the energy of the medicines, targets, and drug-target complexes from complexes’ binding affinities:

Δ*G*_bind_ = Δ*E*_MM_ + Δ*G*_sol_ − TΔ*S* (1)

Δ*E*_MM_ = Δ*E*_internal_ + Δ*E*_electrostatic_ + Δ*E*_vdw_ (2)

Δ*G*_sol_ = Δ*G*_PB_ + Δ*G*_SA_ (3)

where Δ*E*_MM_ stands for the energy of the drug-protein interaction. Additionally, Δ*G*_sol_ is the sum of the polar and non-polar contributions of Δ*G*_PB_ (electrostatic solvent free energy) and Δ*G*_SA_ (non-electrostatic solvent component) to the solvent energy. Since several earlier studies have shown that entropy does not contribute much to the binding free energy to the same protein, the entropy contribution was not calculated in this work [91,92]. 

### 4.7. Cell Culture

Both the human prostate adenocarcinoma LNCaP cell line and the African green monkey kidney cell line CV-1, were acquired from the American Type Culture Collection (ATCC; Manassas, VA, USA). Cells were cultured in RPMI1640 medium or Dulbecco’s Modified Eagle’s Medium (DMEM), respectively, and supplemented with 10% FBS, antibiotics, and 5% CO_2_ at 37 °C. Stable transfected pRC1 CMV-Nur77 antisense LNCaP cells were selected by G418 (Gibco-BRL, Grand Island, NY, USA). Simultaneously, the vector was stably transfected in the same way [63]. 

### 4.8. Cell Proliferation Assay

LNCaP cells were inoculated in a 96-well plate at 3000 cells/well. As described previously [21], after the treatment, the viable cells were determined by 3-(4,5-dimethylthiazol-2yl)-5-(3-carboxymethoxyphenyl)-2-(4-sulfophenyl)-2H-tetrazolium-phenomenon methosulfate (MTT) (Sigma-Aldrich St. Louis, MO, USA) and the absorbance values (OD values) were measured and recorded by the microplate reader. The inhibition ratio was calculated.

### 4.9. Apoptosis Analysis

The process was the same as described previously [21]. Briefly, cells were fixed with 70% ethanol and digested by DNase-free RNase A, then stained using PI, followed by flow cytometry (FCM, Beckman) detection. In addition, the same aliquot of stained cell samples was also examined by fluorescence microscopy for nuclear morphology analysis. 

### 4.10. Western Blot

The process was almost the same as the previous protocol [21]. In brief, sodium dodecyl sulfate-polyacrylamide gel electrophoresis (SDS–PAGE) was utilized to separate the cell lysates. After that, proteins in the gel were transferred to the nitrocellulose (NC) membrane, which was blocked in a blocking solution (5% non-fat milk in TBS/T buffer). This was followed by incubation with diluted primary and secondary antibodies in a blocking buffer. An ECL substrate was used for the detection of the signal.

### 4.11. Chloramphenicol Acetyltransferase (CAT) Reporter Assay

Transient transfection was performed using a modified calcium phosphate precipitation procedure [93,94]. In the presence or absence of Nur77/RXRα expression vector, β-galactosidase expression vector along with chloramphenicol acetyltransferase (CAT), and carrier DNA was transiently transfected into LNCaP or CV-1 cells. As required, cells were treated for 24 h, using malayoside, SR11237, or 9-cis RA. The cells were then collected to determine CAT and β-galactosidase activities.

### 4.12. Subcellular Fractionation

As in our previous report, the cells were suspended in a hypotonic buffer [21], containing proteinase inhibitors and homogenized. After centrifugation, the pellet was resuspended in 1.6 M sucrose, laying over 2.0 M sucrose, in a hypotonic buffer plus protease inhibitors. The nuclear fraction was obtained by centrifuge at 150,000× *g*. The heavy membrane (HM) and cytosolic fractions were obtained by the centrifugation of the supernatants. Both nuclear and HM fractions were lysed by a lysis buffer.

### 4.13. Cell Transfections for Overexpression

Cells were incubated overnight and then transfected with the plasmid pCMV-Myc-Nur77 or GFP-Nur77 [22,72] by Lipofectamine 2000.

### 4.14. Confocal Fluorescent Microscopy

Cells were plated onto coverslips before the treatment with malayoside, then fixed at room temperature using 4% paraformaldehyde. After being permeabilized with 0.1% Triton X-100, the cells were blocked and reacted with the primary antibody, then continued byCy3 labeled anti-mouse IgG or anti-goat IgG conjugated with Alexa fluor 488. The nuclear localization was visualized by DAPI staining.

### 4.15. Statistical Analysis

Differences between groups were compared by a Student’s *t*-test using Prism 5 (GraphPad, San Diego, CA, USA). The data are shown as mean ± SEM. 

## 5. Conclusions

In this study, we used a bioinformatics analysis to explore whether Nur77 might be a useful prognostic marker for PRAD patients and if it could be a marker for the change of TIME. Moreover, Nur77 could be a target for PRAD. The molecular mechanism of malayoside in PRAD cells has been mapped, which included the NRs and MAPKs signaling pathways. Malayoside may target Nur77 to induce PRAD cell apoptosis and to affect immune infiltration. However, further studies are necessary to elucidate Nur77 expression levels and subcellular localization with tumor-infiltrating cells in PRAD patients. The concise mechanism, especially the exact signaling pathway, will be determined in our future study. 

## Figures and Tables

**Table 1:** The binding free energy and its components for the malayoside with target Nur77.

Components	Mean (kJ/mol)	Std (kJ/mol)
Δ*E*_vdwz_	−158.824	3.268
Δ*E*_electrostatic_	−31.491	5.562
Δ*G*_PB/GB_	117.443	9.561
Δ*G*_SA_	−15.794	0.300
Δ*G*_bind_	−88.856	4.852

Δ*E*_vdw_, van der Waals energy; Δ*E*_electrostatic_, electrostatic energy; ΔG_PB_, the polar solvation energy with the PB model; Δ*G*_bind_ is the sum of Δ*E*_vdw_ + Δ*E*_electrostatic_ + Δ*G*_PB/GB_ + Δ*G*_SA_.

## Data Availability

Data will be made available if required.

## Sample Availability

Samples of malayoside are available from the authors.
